# The Serine Shunt enables formate conversion to formaldehyde *in vivo*

**DOI:** 10.1093/sumbio/qvae024

**Published:** 2024-08-31

**Authors:** Karin Schann, Sebastian Wenk

**Affiliations:** Max Planck Institute of Molecular Plant Physiology, Am Mühlenberg 1, 14476 Potsdam-Golm, Germany; Max Planck Institute of Molecular Plant Physiology, Am Mühlenberg 1, 14476 Potsdam-Golm, Germany

**Keywords:** formate assimilation, formate reduction, formaldehyde assimilation, synthetic C1 metabolism, growth-coupled design

## Abstract

Microbial valorization of CO_2_-derived substrates has emerged as a promising approach to address climate change and resource scarcity. Formate, which can be efficiently produced from CO_2_, shows great potential as a sustainable feedstock for biotechnological production. However, the scope of formate assimilation pathways is restricted by the limited number of natural formate-assimilating enzymes. To overcome this limitation, several new-to-nature routes for formate assimilation based on its reduction to formaldehyde have been proposed, but they suffer from low catalytic efficiencies and cannot yet support bacterial growth. Here, we propose the Serine Shunt as a novel formate reduction route and demonstrate its activity *in vivo*. In this pathway, formate is attached to glycine to form serine, which is subsequently cleaved into formaldehyde and glycine, thereby effectively converting formate to formaldehyde. Unlike other formate reduction routes, the Serine Shunt mainly utilizes natural reactions with favorable enzyme kinetics, while requiring the same amount of ATP and NADPH as the most efficient new-to-nature route. We implemented the Serine Shunt in engineered *Escherichia coli* strains using a stepwise approach by dividing the pathway into metabolic modules. After validating the individual module activities, we demonstrated the *in vivo* activity of the complete Serine Shunt by measuring intracellular formaldehyde production with a green fluorescent protein (GFP) sensor and coupling its activity to cell growth. Our results indicate that the Serine Shunt could be applied as a novel formate reduction route in methylotrophic hosts relevant for biotechnology.

**Sustainability Statement**

The research presented in this article contributes to advancing sustainability by addressing critical global challenges associated with climate change and resource scarcity. Specifically, the development and implementation of the Serine Shunt as a novel pathway for formate reduction align with several United Nations Sustainable Development Goals:

Goal 9: Industry, Innovation, and Infrastructure—The introduction of the Serine Shunt enhances the biotechnological applications of microbial systems. This innovation facilitates the development of sustainable industrial processes by improving the efficiency of microbial production systems. The use of engineered *Escherichia coli* strains to implement the pathway demonstrates a practical approach to sustainable industrial biotechnology.

Goal 13: Climate Action—By focusing on the microbial assimilation of CO_2_-derived substrates, this research directly contributes to climate action efforts. The conversion of CO_2_ into useful chemicals not only helps to mitigate greenhouse gas emissions but also promotes the recycling of carbon, thereby reducing the overall carbon footprint of industrial processes.

In conclusion, the Serine Shunt represents a significant advancement in microbial biotechnology with the potential to transform CO_2_ utilization and reduce environmental impact. The alignment with the United Nations Sustainable Development Goals underscores the importance and relevance of this research in promoting a more sustainable future.

## Introduction

The rapidly progressing climate change requires immediate actions to reduce the CO_2_ concentration in the atmosphere (Lee et al. 2022). This should be addressed by reducing CO_2_ emissions and by capturing CO_2_ from the atmosphere, followed by its storage or valorization. Biotechnological valorization of captured CO_2_ is a promising alternative to long-term storage as it recycles CO_2_ and minimizes the need for fossil or plant-derived products (Bachleitner et al. 2023). Here, reduced one-carbon (C1) molecules such as formate or methanol could become important mediators as they can be electrochemically produced from CO_2_ and present favorable characteristics for microbial cultivation. In recent years, the field of C1 biotechnology has emerged as a subdiscipline that aims to combine chemical CO_2_ reduction with biotechnological conversion of C1 molecules to value-added products (Bachleitner et al. 2023, Orsi et al. 2023). The model of a formate-based bioeconomy proposes to explore formate as a favorable C1 substrate for the sustainable biotechnology (Yishai et al. 2016).

In nature, several bacteria grow aerobically on formate via the serine cycle (Anthony 2011). Here, formate is assimilated into methylene-THF (CH_2_-THF), which is condensed with glycine to form serine. Thereafter, a chain of reactions leads to the recycling of glycine and the production of glyoxylate as a biomass precursor. Based on the formate assimilation module of the serine cycle, several new-to-nature metabolic pathways for formate assimilation in model microbes were suggested (Bar-Even et al. 2013). In recent years, two of these pathways, the reductive glycine pathway and the serine threonine cycle, have been successfully engineered in model microbes (Bang et al. 2020, Claassens et al. 2020, Kim et al. 2020, Turlin et al. 2022, Wenk et al. 2022). However, the growth characteristics of the engineered microbes are still suboptimal for biotechnological applications. Thus, growth on formate via these pathways should be improved, or other formate assimilation routes should be explored.

To increase the number of formate assimilation routes, formate could be converted to formaldehyde, which is a more reduced C1 mediator with higher energy density. Furthermore, it is more reactive than formate and can be easily assimilated into metabolism. It has the same reduction level as biomass and many formaldehyde assimilation reactions exist (both natural and synthetic) (Bar-Even 2016). For example, many methylotrophic bacteria and yeasts, relevant for biotechnology, grow on methanol-derived formaldehyde via the ribulose monophosphate cycle and the xylulose monophosphate cycle (Chistoserdova et al. 2009, Pham et al. 2022, Wegat et al. 2022, Wefelmeier et al. 2023). Furthermore, several promising new-to-nature pathways for formaldehyde assimilation such as the homoserine cycle, the formolase pathway, the synthetic acetyl-CoA (SACA) pathway, and the erythrulose monophosphate (EuMP) cycle have been proposed (Siegel et al. 2015, Lu et al. 2019, He et al. 2020, Wu et al. 2023).

As a metabolic pathway that converts formate to formaldehyde does not seem to exist in nature, two new-to-nature enzymatic cascades were previously proposed: (i) formate activation to formyl-CoA or (ii) formate activation to formyl phosphate followed by reduction of the activated intermediate to formaldehyde (Bar-Even 2016). Although enzyme activities of both cascades could be demonstrated, they were not sufficient to support cellular growth, suggesting that further enzyme optimization is required (Wang et al. 2021, Hu et al. 2022, Nattermann et al. 2023).

**Figure 1.**
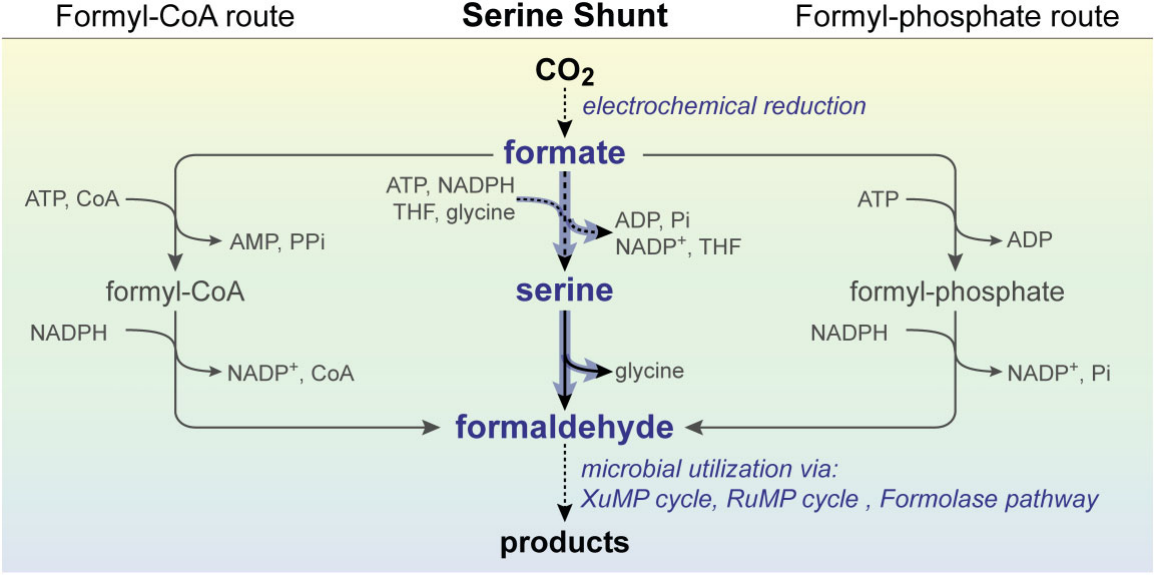
The Serine Shunt compared to other new-to-nature formate reduction routes. Reaction schemes for the different proposed formate reduction routes. The new-to-nature routes are shown in light font. The Serine Shunt is shown in bold font. Dotted arrows indicate multi-enzymatic reactions. Formate can be reduced into formaldehyde via two new-to-nature reaction routes, which use formyl-CoA of formyl phosphate as intermediates. In the Serine Shunt, formate is reduced to formaldehyde via its condensation with glycine to serine followed by serine cleavage to glycine and formaldehyde. The Serine Shunt and the formyl phosphate route are the most energy efficient formate reduction routes, as they consume one ATP and one NADPH to reduce one mole formate.

In this work, we propose the Serine Shunt as an alternative route for formate conversion to formaldehyde (Fig. 1). By leveraging metabolic reactions from different C1 assimilation pathways, we designed a linear route that assimilates formate into serine and releases formaldehyde via serine cleavage. Like in the natural serine cycle, formate is assimilated into CH_2_-THF, which is attached to glycine to form the stable intermediate serine. Thereafter, the serine aldolase from the synthetic homoserine cycle is used to cleave serine into formaldehyde and glycine.

Using a stepwise engineering approach, we divided the Serine Shunt into two metabolic modules and tested the individual module activities in dedicated selection strains. Then, we combined both modules into a strain that depends on formaldehyde assimilation for cell growth and were able to show formate-dependent growth via the novel route. Using ^13^C labeling, we confirmed the conversion of formate to formaldehyde. The Serine Shunt presents a promising route to produce formaldehyde *in vivo* and could be directly applied in metabolic engineering studies aiming to establish novel formate assimilation pathways.

## Materials and methods

### Chemicals and reagents

Primers were synthesized at Integrated DNA Technologies (IDT). DNA fragments were amplified with the high-fidelity polymerase PrimeSTAR Max DNA Polymerase from TaKaRa and colony PCR reactions were performed using DreamTaq polymerase (Thermo Fisher Scientific). FastDigest restriction enzymes and T4 DNA ligase from Thermo Fisher Scientific were used for restriction-based cloning. The kits used for plasmid isolation and PCR cleanup were purchased from Thermo Fischer Scientific. All chemicals used in the plate reader experiments were ordered from Sigma–Aldrich.

### Growth media

For cloning and strain engineering, cultures were grown in lysogeny broth (LB) medium (10 g/l NaCl, 10 g/l Tryptone, 5 g/l yeast extract) supplemented with the relevant antibiotic (50 μg/ml kanamycin or 100 μg/ml streptomycin). Diaminopimelate (DAP, 0.25 mM) needs to be supplemented to the growth media of the formaldehyde sensor due to the *asd* knockout. During growth experiments, cells were cultivated in M9 minimal medium (50 mM Na_2_HPO_4_, 20 mM KH_2_PO_4_, 1 mM NaCl, 20 mM NH_4_Cl, 2 mM MgSO_4_, and 100 μM CaCl_2_), supplemented with trace elements (134 μM EDTA, 31 μM FeCl_3_, 6.2 μM ZnCl_2_, 0.76 μM CuCl_2_, 0.42 μM CoCl_2_, 1.62 μM H_3_BO_3_, 0.081 μM MnCl_2_) and the relevant carbon sources (see the “Growth experiments” section).

### Strains

Plasmid construction was done in *Escherichia coli* DH5α. The *E. coli* SIJ488 strain, which is derived from K-12 MG1655, was used as a base strain (Jensen et al. 2015). The KEIO collection was used as donor strains to prepare the P1 donor lysates for phage transduction (Baba et al. 2006).

### Strain engineering

The sensor strains were constructed by using recombineering techniques to modify the SIJ488 base strain. Deletions were made by P1 phage transduction, as detailed in Wenk et al. (2018). For P1 phage transduction, the KEIO collection strain with the desired knockout was grown in LB supplemented with 5 mM CaCl_2_ until an OD of ∼0.3. Then, the culture was infected with P1vir (Ikeda and Tomizawa 1965) stock lysate for 2–4 h. The cells were pelleted by centrifugation and the supernatant was filtered through a 0.22-μm filter to obtain the donor lysate that was then used for the transduction. The deletion in the relevant strain was made by resuspending the pellet of an overnight culture in P1 salts (LB supplemented with 10 mM CaCl_2_ and 5 mM MgSO_4_). P1 donor lysate was added to this cell suspension and incubated for 30 min at 30°C. After a recovery for 1 h in LB with ∼100 mM sodium citrate, the cells were plates on selective plates containing 5 mM sodium citrate and kanamycin. The antibiotic cassette was later removed by inducing the flippase. Knockouts and removal of the antibiotic cassette were confirmed by a colony PCR.

Genomic integrations were made using the recombineering machinery of the SIJ488 strain (Jensen et al. 2015). Therefore, the gene of interest was cloned together with a kanamycin antibiotic cassette, flanked by FRT sites, and amplified with primers containing 50-bp homology arms that are flanking the genomic integration spot. This linear DNA fragment was introduced in the cell by electroporation after 1 h of inducing the λ-Red recombineering machinery with 15 mM arabinose. The cell suspension was then plated on LB plates containing the relevant antibiotics and a colony PCR was performed to confirm successful gene deletions. The antibiotic cassettes were removed by inducing the flippase with 50 µM l-rhamnose for at least 4 h and removal was confirmed by a colony PCR. M2 was integrated into the genomic safe spot 9 (Bassalo et al. 2016) using a kanamycin marker and expressed under the control of a strong constitutive promoter (P_pgi-20_) and a medium-strength RBS (RBS_C_). To create the formaldehyde sensor, the gene *rhmA* from *E. coli* that encodes for the HAL reactions was integrated into the genomic safe spot 2 (Bassalo et al. 2016) using a kanamycin marker under the control of P_pgi-20_ and RBS_C_. Strains used in this study are listed in Table 1.

**Table 1.** Strains used in this study.

Strain name	Gene deletions	Gene insertion	Plasmid	Source	Used in figure
SerAux	∆*gcvTHP* ∆*serA*	None	None	This study	Supplmentary Fig. S1
SerAux	∆*gcvTHP* ∆*serA*	None	pM1	This study	Supplmentary Fig. S1
GFP sensor	∆*gcvTHP* ∆*serA* ∆*frmRAB*	None	pTR47m4-GFP	This study	Fig. 3
GFP sensor + M2	∆*gcvTHP* ∆*serA* ∆*frmRAB*	SS9:P_pgi-20_:RBS_C_:ltaE	pTR47m4-GFP	This study	Fig. 3
GFP sensor + M1 + M2	∆*gcvTHP* ∆*serA* ∆*frmRAB*	SS9:P_pgi-20_:RBS_C_:ltaE	pTR47m4-GFP pM1	This study	Fig. 3
Serine Shunt strain	∆*gcvTHP* ∆*serA* ∆*frmRAB* ∆*asd*	SS2:P_pgi-20_:RBS_C_:rhmA	None	This study	Fig. 4
Serine Shunt strain + M1	∆*gcvTHP* ∆*serA ∆frmRAB ∆asd*	SS2:P_pgi-20_:RBS_C_:rhmA	pM1	This study	Fig. 4
Serine Shunt strain + M1 + M2	∆*gcvTHP* ∆*serA* ∆*frmRAB* ∆*asd*	SS2:P_pgi-20_:RBS_C_:rhmA, SS9:P_pgi-20_:RBS_C_:ltaE	pM1	This study	Figs 4 and 5, and Supplmentary Fig. S2

### Synthetic operon construction

All genes were codon optimized for *E. coli* K-12, after which a 6xHis tag was added to the N-terminal side. Subsequently, the genes were inserted into a cloning vector, which attaches RBS_C_ upstream of the gene (Zelcbuch et al. 2013). These cloning vectors were used to assemble synthetic operons via restriction and ligation using the protocol as described in Wenk et al. (2018). The assembled operon was then cloned into an expression vector pZ (p15A origin of replication, streptomycin marker) under the control of P_pgi-20_ (Wenk et al. 2018). pTR47m4-GFP (Woolston et al. 2018) was used to construct the GFP sensor. Plasmids used in this study are listed in Table 2.

**Table 2.** Plasmids used in this study.

Plasmid name	Genes	Source	GeneBank ID
pTR47m4-GFP		Woolston et al. (2018)	
pM1	p15A-ori; Strep^R^, P_pgi-20_:RBS_C_:*ftfL-fch-mtdA-glyA*	This study	*ftfL* Q83WS0 *fchA Q49135* *mtdA* P55818 *glyA* P0A825

### Growth experiments

Prior to all growth experiments, the strains were streaked out from the glycerol stock on LB plates (supplemented with the respective antibiotic and 0.25 mM DAP in the case of the formaldehyde sensor) and grown overnight at 37°C. From these plates, precultures in relaxing liquid M9 medium (as indicated in Table 3) were inoculated for overnight growth at 37°C and 220 rpm. The cells were harvested (8000 rpm, 3 min), washed three times with M9 minimal medium, and diluted to a final OD_600_ of 0.01 in the respective medium. In a Nunc 96-well microplates (Thermo Fisher Scientific), 150 µl of the culture was added to each well, and the wells were covered with 50 µl of mineral oil (Sigma–Aldrich) to prevent evaporation. Growth experiments were performed in the BioTek Epoch2 plate reader (BioTek Instrument, USA), which measured the optical density after each kinetic cycle. A cycle consists of 12 shaking steps in which 60 s linear and 60 s orbital shaking were alternated (1 mm amplitude). The growth experiments that required fluorescence measurement were performed in a Tecan Infinite 200 Pro plate reader (Tecan, Switzerland). OD_600_ values measured in the plate reader were calibrated to represent OD_600_ values in standard cuvettes according to OD_cuvette_ = OD_plate_/0.23. The mean of the technical duplicates is shown in the growth curves and the raw data are provided in the Supplementary Data file.

**Table 3.** Composition of media for growth experiments.

Used for	SerAux strain	GFP sensor	Serine Shunt strain
Preculture medium	10 mM glucose 1 mM serine	10 mM glucose 1 mM serine	10 mM glucose 1 mM isoleucine 1 mM threonine 0.25 mM DAP 1 mM methionine
Figs 3A and 4A	10 mM glucose 1 mM serine	10 mM glucose 1 mM serine	10 mM glucose 1 mM isoleucine 1 mM threonine 0.25 mM DAP 1 mM serine
Figs 3B and 4B	10 mM glucose 1 mM glycine 1 mM sodium formate	10 mM glucose 1 mM glycine 1 mM sodium formate	10 mM glucose 1 mM isoleucine 1 mM threonine 0.25 mM DAP 1 mM glycine 4 mM sodium formate

### Labeling of proteinogenic amino acids

For stationary isotope tracing of proteinogenic amino acids, cells were cultured in 4 ml of M9 medium with all required carbon sources and supplements (10 mM glucose, 1 mM isoleucine, 1 mM threonine, 0.25 mM DAP, 1 mM glycine) as well as either labeled or unlabeled 4 mM sodium formate. After reaching the stationary phase, the cells were collected by centrifugation for 3 min at 8000 rpm. Biomass was hydrolyzed by incubation with 1 ml of 6 N hydrochloric acid for a duration of 24 h at 95°C. Samples were dried via heating at 95°C and re-dissolved in 1 ml of ddH_2_O. Hydrolyzed amino acids were separated using ultra-performance liquid chromatography (Acquity, Waters) using a C18-reversed-phase column (Waters) as previously described in Giavalisco et al. (2011). Mass spectra were acquired using an Exactive mass spectrometer (Thermo Fisher). Data analysis was performed using Xcalibur (Thermo Fisher). Before analysis, amino acid standards (Sigma–Aldrich) were analyzed under the same conditions to determine typical retention times. The experiment was performed in biological duplicates, of which only the results of biological replicate 2 are shown in Fig. 5B. The results for both biological replicates are shown in Supplementary Fig. S2.

## Results

### The metabolic architecture of the Serine Shunt

**Figure 2.**
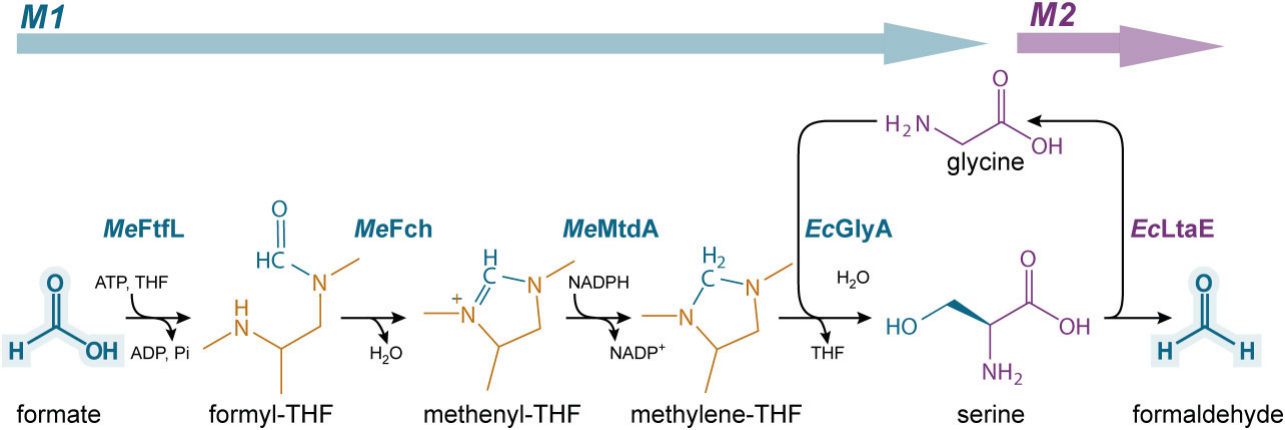
Molecular architecture of the Serine Shunt. The Serine Shunt can be divided into two modules: M1 (blue arrow + enzyme names) and M2 (purple arrow + enzyme names). In M1, formate (blue) is attached to tetrahydrofolate (THF) (orange), forming a C1-THF intermediate, which is reduced to methylene-THF. The reduced C1 is subsequently transferred from THF onto glycine (purple), resulting in the formation of serine. In M2, serine gets cleaved, releasing the C1 moiety as formaldehyde and recovering glycine. THF, tetrahydrofolate; *Me*FtfL, formate THF ligase; *Me*Fch, methenyl-THF cyclohydrolase; *Me*MtdA, methylene-THF dehydrogenase; *Ec*GlyA, serine hydroxymethyltransferase; and *Ec*LtaE, threonine aldolase.

We designed the Serine Shunt based on the formate assimilation reactions of the serine cycle and the serine aldolase reaction of the homoserine cycle (Anthony 2011, He et al. 2020). To facilitate the testing of the shunt, we divided it into two metabolic modules (Fig. 2). Module 1 (M1) converts formate and glycine to serine and module 2 (M2) cleaves serine to formaldehyde and glycine. In M1, formate undergoes a series of enzymatic transformations catalyzed by *Methylorubrum extorquens’* formate THF ligase (*Me*FtfL), methenyl-THF cyclohydrolase (*Me*Fch), and methylene-THF dehydrogenase (*Me*MtdA), consuming ATP and NADPH. The resulting methylene-THF then combines with glycine via *E. coli*’s serine hydroxymethyltransferase (*Ec*GlyA) to form serine. In M2, serine is cleaved into formaldehyde and glycine via the serine aldolase reaction catalyzed by the threonine aldolase from *E. coli* (*Ec*LtaE). Notably, glycine can be considered a catalytic intermediate that functions as a methyl group carrier without being consumed by the pathway.

### Testing of pathway modules confirms *in vivo* activity of the Serine Shunt

While both modules of the Serine Shunt have been used in other metabolic context (He et al. 2020, Kim et al. 2020, Wenk et al. 2022), we assessed their individual activity first, before combining them into the complete shunt. To test M1, we introduced a plasmid expressing the module into a serine auxotrophic *E. coli* strain deleted in the phosphoglycerate dehydrogenase (Δ*serA*) and the glycine cleavage system (Δ*gcvTHP*) (SerAux strain) (Supplementary Fig. S1A). While Δ*serA* creates a serine auxotrophy, which allows to select for formate conversion into serine, Δ*gcvTHP* prevents methylene-THF formation from glycine, ensuring dependency on formate assimilation. This strain was only capable of growing on glucose and glycine when we expressed M1 and supplemented formate (Supplementary Fig. S1B and C).

**Figure 3.**
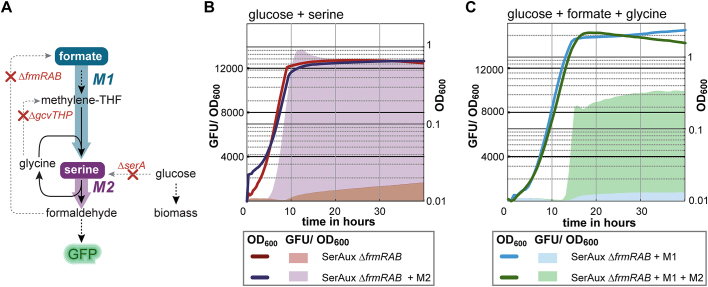
Testing of Serine Shunt modules in the GFP sensor. (A) Metabolic scheme of the GFP sensor. Gene deletions are indicated as red crosses. Multi-enzymatic reactions are shown as dotted arrows. As before, M1 is shown in blue color and M2 in purple. (B) M2 activity was tested in the GFP sensor, cultivated on glucose as biomass precursor and serine as formaldehyde precursor. The green fluorescence normalized to the OD_600_ (GFU/OD_600_) is shown on the left y-axis (shadow area) and the OD_600_ (curve) on the right one. Despite similar growth phenotypes between the GFP sensor with M2 overexpression (purple curve) and without (orange curve), only the strain overexpressing M2 produced a fluorescent signal (purple shadow). (C) The complete Serine Shunt was tested in the GFP sensor was cultivated on glucose as biomass source, glycine as catalyzing intermediate, and formate as formaldehyde source. Through M1 expression, formate could be converted to serine, releasing the strain’s auxotrophy (blue curve). Only upon expression of both modules, green fluorescence could be measured, indicating the activity of the complete Serine Shunt. *frmRAB*, formaldehyde detoxification system; *gcvTHP*, glycine cleavage system; *serA*, serine hydroxymethyltransferase.

Subsequently, we assessed whether *Ec*LtaE can produce formaldehyde from serine *in vivo* and thus can be harnessed as M2 of the Serine Shunt. To this end, we deleted the formaldehyde detoxification system (Δ*frmRAB*) in the SerAux strain to prevent formaldehyde oxidation to formate. Subsequently, we introduced a formaldehyde-sensing plasmid (developed by Woolston et al. 2018) that allows to detect intracellular formaldehyde, by generating a GFP signal upon formaldehyde detection and referred to the resulting strain as GFP sensor (Fig. 3A). To test M2 activity in this strain, we cultivated it on glucose and serine (Fig. 3B). Only when *Ec*LtaE was overexpressed a GFP signal could be detected, confirming the enzyme’s ability to catalyze the conversion of serine into formaldehyde and its suitability as M2 of the Serine Shunt.

Having confirmed the individual activities of M1 and M2, we proceeded to evaluate their combined activity. To this end, we expressed both modules in the GFP sensor. We cultivated the resulting strain on glucose, glycine, and formate and measured the GFP signal over time. While the strain was able to grow when M1 was expressed alone (release of the serine auxotrophy), only when both modules were expressed together a GFP signal was detected (Fig. 3C). These results demonstrated for the first time the activity of the complete Serine Shunt. The delay in GFP signal observed in Fig. 3C can likely be attributed to the availability of serine, which is cleaved by *Ec*LtaE to glycine and formaldehyde. When serine is provided in the medium, it is immediately cleaved, leading to an FA signal shortly after growth begins. In contrast, when formate and glycine are provided in the medium, serine must first be enzymatically produced and is continually consumed during growth. This results in a delayed GFP signal.

### The Serine Shunt carries sufficient flux to support cell growth

**Figure 4.**
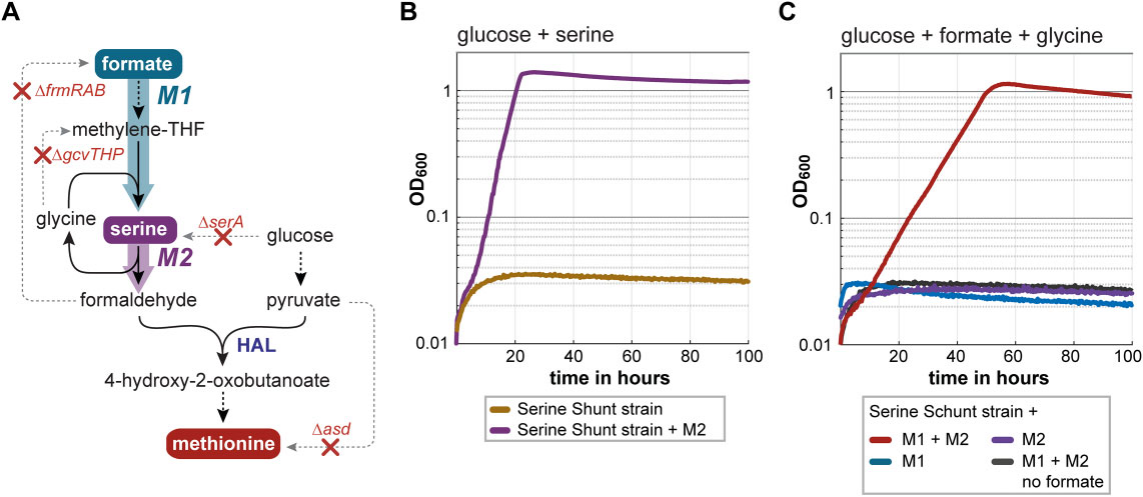
The Serine Shunt supports formate-dependent growth. (A) The metabolic scheme of the formaldehyde sensor, which was used to test the complete Serine Shunt. The methionine auxotrophy is shown as a red circle. Gene deletions are visualized with a red cross. Multi-enzymatic reactions are indicated by dotted arrows. M1 is visualized in blue color, whereas M2 is shown in purple. (B) The Serine Shunt strain was used to test M2 activity. M2 overexpression (purple curve) is sufficient to enable the strain growth on glucose (pyruvate precursor) and serine (formaldehyde precursor). (C) Combining both modules in the Serine Shunt strain (red curve) enables growth on glucose (pyruvate precursor), formate (formaldehyde precursor), and glycine (catalyzing agent). *asd*, aspartate-semialdehyde dehydrogenase; *frmRAB*, formaldehyde detoxification system; *gcvTHP*, glycine cleavage system; *serA*, serine hydroxymethyltransferase; HAL, HOB aldolase.

While the activity of the Serine Shunt could be demonstrated using the GFP sensor, it is essential to note that this sensor does not use formaldehyde as a source of biomass. To test whether the Serine Shunt can carry sufficient flux to support cell growth, we engineered the SerAux∆*frmRAB* strain into a formaldehyde growth biosensor. Leveraging the design of the HOB biosensor from a previous study (Schann et al. 2023), we deleted the aspartate-semialdehyde dehydrogenase gene (Δ*asd*) and overexpressed the HOB aldolase (HAL) in the SerAux∆*frmRAB* strain creating the Serine Shunt strain. Δ*asd* rendered the strain auxotrophic to various essential amino acids, including methionine, while HAL expression enabled the assimilation of formaldehyde and pyruvate into the methionine precursor 4-hydroxy-2-oxobutanoate (HOB), which is further converted to methionine, releasing the strain’s auxotrophy in the presence of formaldehyde (Fig. 4A).

We cultivated the Serine Shunt strain overexpressing M2 on glucose and serine to test whether serine cleavage by M2 could release the methionine auxotrophy. Only when M2 was overexpressed, the strain’s growth could be rescued, indicating that M2 can produce sufficient formaldehyde for biomass formation (Fig. 4B). To test the complete Serine Shunt in a growth-dependent context, we expressed both modules in the Serine Shunt strain. The resulting strain was tested in a medium supplemented with glucose glycine and formate, requiring formate reduction to formaldehyde for releasing the methionine auxotrophy of the strain. As shown in Fig. 4C, only when both modules were expressed, the strain’s growth was restored, indicating that the Serine Shunt is active *in vivo* and can convert formate into formaldehyde in an amount sufficient for biomass formation.

### Carbon tracing confirms formate conversion to formaldehyde via the Serine Shunt

**Figure 5.**
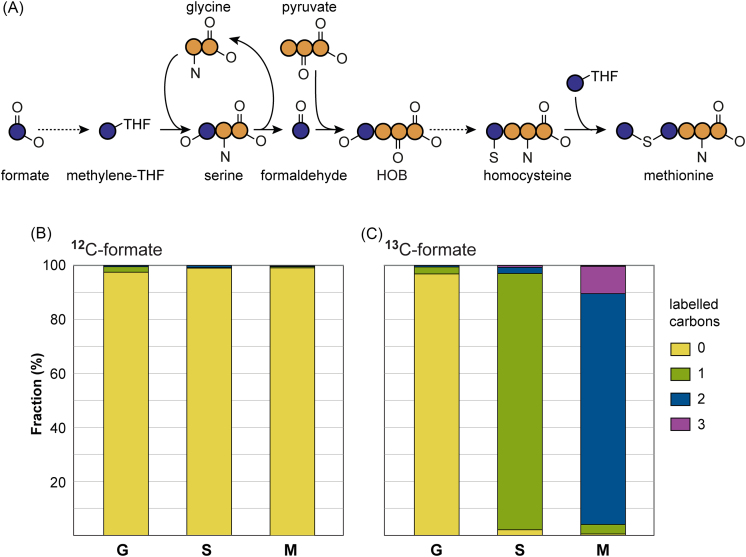
^13^C labeling confirms *in vivo* activity of the Serine Shunt. (A) Expected labeling of proteinogenic amino acids upon cultivation on ^13^C-formate. The carbon derived from formate is colored blue, whereas carbons from all other sources are colored orange. Multi-enzymatic reactions are indicated by dotted arrows. (B) The strain fed with ^12^C-formate shows no labeling in glycine (G), serine (S), and methionine (M). (C) When ^13^C-formate was used as formaldehyde precursor, serine is labeled once (green) and methionine is labeled twice (blue). HOB, 4-hydroxy-2-oxobutanoate.

To confirm that formaldehyde is produced from formate via the Serine Shunt, we used ^13^C labeling to track the metabolic incorporation of the formate-derived carbon in the Serine Shunt strain. In theory, carbon from formate is incorporated into serine via M1 and then cleaved into formaldehyde via M2. In this process, glycine acts solely as a catalytic intermediate and does not mix with the formate-derived carbon. Following this logic, serine should be labeled once, while glycine should remain unlabeled. Next, serine-derived formaldehyde condenses with pyruvate to form HOB, which gets converted into homocysteine. Homocysteine condenses with formate-derived methylene-THF forming methionine. Thus, methionine should be labeled twice (Fig. 5A).

We cultivated the Serine Shunt strain expressing both modules on glucose, glycine, and either non-labeled formate or ^13^C-labeled formate (Fig. 5B). Then, we extracted the proteinogenic amino acids and analyzed their labeling pattern via liquid chromatography–mass spectrometry (LC–MS). When ^13^C-formate was supplied in the medium, all amino acids were labeled as expected. Serine was labeled once, confirming M1 activity. Methionine showed two labeled carbons, confirming the activity of the complete Serine Shunt. Thus, the ^13^C labeling results unequivocally confirm the *in vivo* activity of the Serine Shunt.

## Discussion

The quest for a sustainable, CO_2_-based bioeconomy has driven extensive research efforts in recent years (Bachleitner et al. 2023). While CO_2_ and other C1 gases such as carbon monoxide and methane could be envisioned as substrates for bacterial cultivation, liquid feedstocks such as methanol and formate present multiple advantages ranging from their relative ease of handling to better mass transfer rates (Cotton et al. 2020). Of the liquid substrates, formate can be produced with high efficiencies in a one-step reaction from CO_2_. Thus, formate is considered a prime candidate feedstock for a CO_2_-based bioeconomy (Claassens et al. 2019). Until now, a formate bioeconomy has been postulated and several microbes have been engineered to use formate as a feedstock (Bang et al. 2020, Claassens et al. 2020, Kim et al. 2020, Turlin et al. 2022, Wenk et al. 2022, Bruinsma et al. 2023, Bysani et al. 2024).

An undesired fact that limits the potential of a formate bioeconomy is the limited number of formate-fixing enzymes and the associated small number of metabolic pathways that can be designed from them (Bar-Even 2016). A “metabolic bridge” or “metabolic bypass” (Orsi et al. 2022) that connects formate to formaldehyde would vastly increase the potential of a formate bioeconomy as it would allow to convert methylotrophs into formatotrophs. As enzymatic formate reduction to formaldehyde does not seem to exist in nature, new-to-nature metabolic pathways for this conversion need to be established. This could be achieved by novel enzyme chemistries as suggested for the formyl-CoA and the formyl phosphate route as detailed in Bar-Even (2016) or by metabolic rewiring as proposed for the Serine Shunt. The latter has the advantage of using established enzyme activities that do not require substantial enzyme engineering.

In this work, we present the Serine Shunt as a promising alternative to the previously proposed formate reduction routes. The Serine Shunt uses the formate assimilation module of the natural serine cycle (M1) to convert formate and glycine into serine and the formaldehyde assimilation module of the homoserine cycle (M2) alas in reverse direction for cleaving serine to formaldehyde and glycine. In the Serine Shunt, glycine can be considered as a “catalyzing intermediate,” as it is essential to catalyze the reactions (it serves as a methyl group carrier) but is not consumed by the pathway. While longer in terms of enzymatic reactions, the Serine Shunt resembles previously proposed formate reduction routes in its structure and consumes the same amount of ATP and NADPH as the most energy efficient formyl phosphate route (Bar-Even 2016, Nattermann et al. 2023). Furthermore, it only uses native enzymes allowing immediate implementation without enzyme engineering.

By dividing the pathway into two metabolic modules and coupling module activity to cell growth (growth-coupled selection), we showed that the individual modules are sufficiently active to support cell growth. Our results demonstrated for the first time that the serine aldolase used for formaldehyde assimilation in the synthetic homoserine cycle (He et al. 2020) can efficiently cleave serine to glycine and formaldehyde *in vivo*.

Next, we combined both modules into the complete Serine Shunt and achieved sufficient flux to generate essential cellular building blocks from formate. These results demonstrate that the Serine Shunt is a promising route to establish cell growth via formate reduction.

While in this work only parts of cellular biomass were derived from formate, in future studies the Serine Shunt could be tested in methylotrophs that grow via formaldehyde assimilation pathways such as the RuMP cycle. Here, the Serine Shunt could serve as a metabolic route that generates all biomass from formate, paving the way to a sustainable, CO_2_-based formate bioeconomy.

## Supplementary Material

qvae024_Supplemental_Files

## Data Availability

Additional information on the experimental setup and detailed results are available from the corresponding author upon request. Any strains and plasmids generated during this study are available upon completing a Materials Transfer Agreement.
