# Supplementary material for: The Serine Shunt enables formate conversion to formaldehyde in vivo
Source: Sustain Microbiol. 2024 Aug 31;1(1):qvae024. doi: 10.1093/sumbio/qvae024 (PMC13375380; doi:10.1093/sumbio/qvae024)
Supplement: Supplementary file 1 [file qvae024_Supplemental_Files.zip › Supplementary Figures.pdf]

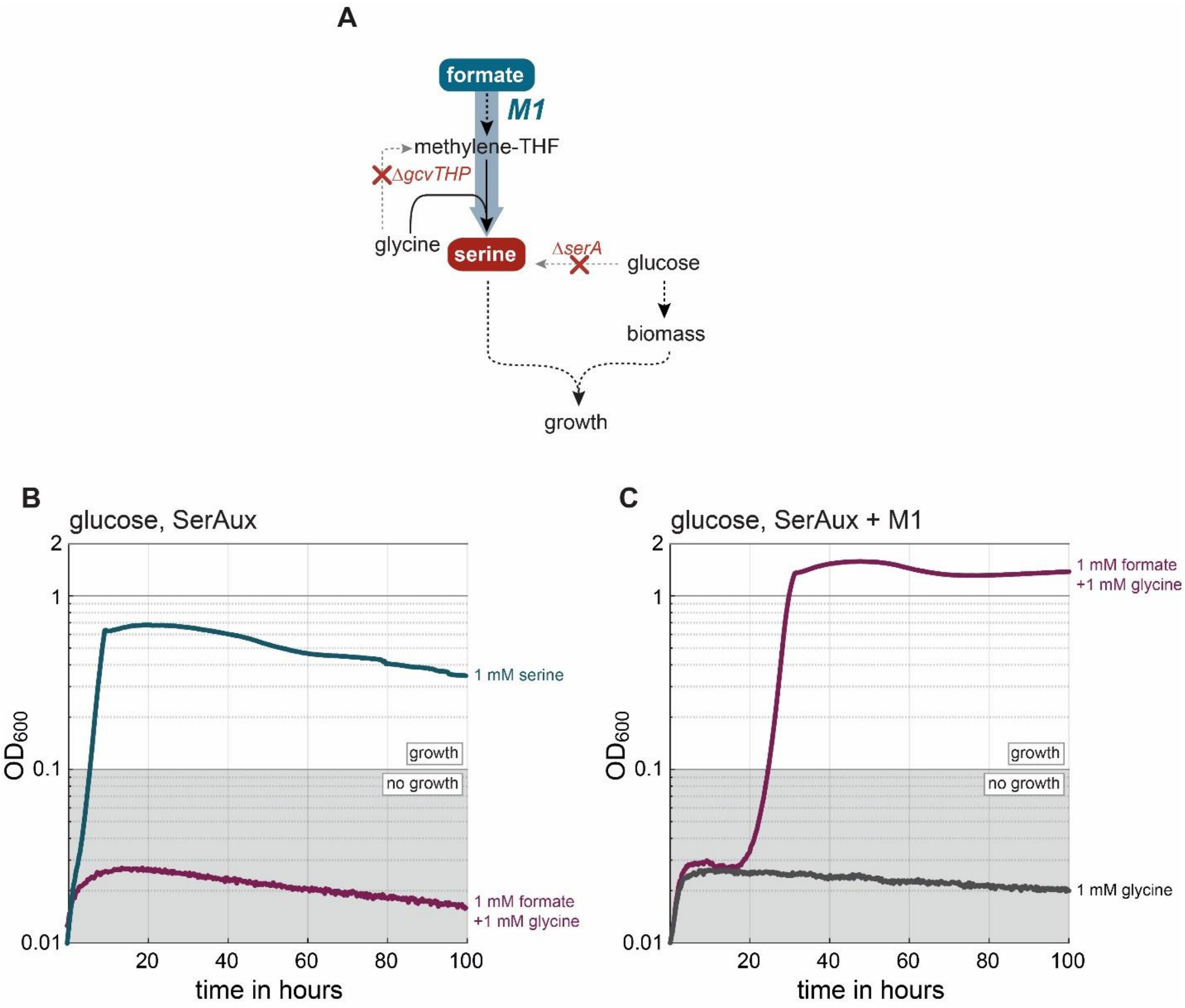

Supplementary Figure 1: Testing M1 activity in SerAux strain

(A) Metabolic scheme of the SerAux sensor. Gene deletions are indicated as red crosses. Multi-enzymatic reactions are shown as dotted arrows. M1 is shown in blue color. (B) The SerAux strain is only capable to grow on glucose when serine is supplemented additionally (blue curve). (C) M1 activity was tested in the SerAux sensor, cultivated on glucose as biomass precursor and formate and glycine as serine precursor. Through M1 expression, formate and glycine could be converted to serine, releasing the strains' auxotrophy (purple curve). *gcvTHP* glycine cleavage system, *serA* serine hydroxymethyltransferase.

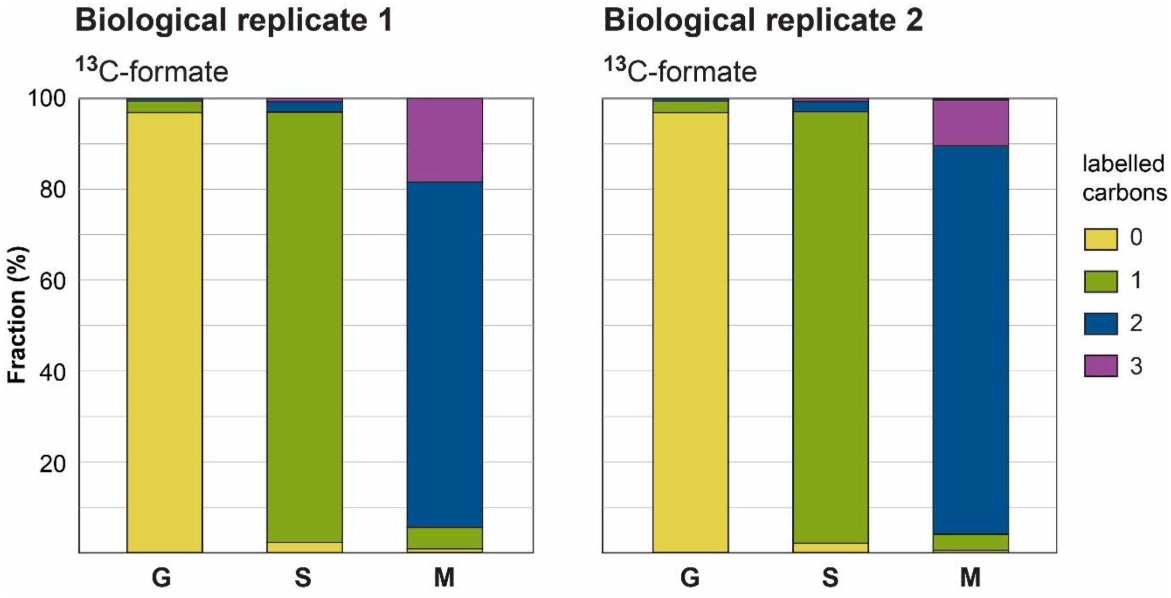

Supplementary Figure 2: ^{13}C labelling data

The results of the ^{13}C labelling experiment are shown for two biological replicates. The strains were cultivated with ^{13}C-labelled formate and the number of labelled carbons in the amino acids: glycine (G), serine (S) and methionine (M), are shown.
